# Longitudinal assessment of ventricular volume trajectories in early-stage schizophrenia: evidence of both enlargement and shrinkage

**DOI:** 10.1186/s12888-024-05749-5

**Published:** 2024-04-24

**Authors:** Patrik Svancer, Vaclav Capek, Antonin Skoch, Miloslav Kopecek, Kristyna Vochoskova, Marketa Fialova, Petra Furstova, Lea Jakob, Eduard Bakstein, Marian Kolenic, Jaroslav Hlinka, Pavel Knytl, Filip Spaniel

**Affiliations:** 1 National Institute of Mental Health, Topolova 748, 250 67 Klecany, Czech Republic; 2 Third Faculty of Medicine, Charles University, Prague, Czech Republic; 3 Department of Diagnostic and Interventional Radiology, Institute for Clinical and Experimental Medicine, Prague, Czech Republic; 4 Department of Cybernetics, Faculty of Electrical Engineering, Czech Technical University in Prague, Prague, Czech Republic

**Keywords:** First-episode schizophrenia, Ventricular volumes, MRI, Longitudinal design, Negative symptoms

## Abstract

**Background:**

Lateral ventricular enlargement represents a canonical morphometric finding in chronic patients with schizophrenia; however, longitudinal studies elucidating complex dynamic trajectories of ventricular volume change during critical early disease stages are sparse.

**Methods:**

We measured lateral ventricular volumes in 113 first-episode schizophrenia patients (FES) at baseline visit (11.7 months after illness onset, SD = 12.3) and 128 age- and sex-matched healthy controls (HC) using 3T MRI. MRI was then repeated in both FES and HC one year later.

**Results:**

Compared to controls, ventricular enlargement was identified in 18.6% of patients with FES (14.1% annual ventricular volume (VV) increase; 95%CI: 5.4; 33.1). The ventricular expansion correlated with the severity of PANSS-negative symptoms at one-year follow-up (*p* = 0.0078). Nevertheless, 16.8% of FES showed an opposite pattern of statistically significant ventricular shrinkage during ≈ one-year follow-up (-9.5% annual VV decrease; 95%CI: -23.7; -2.4). There were no differences in sex, illness duration, age of onset, duration of untreated psychosis, body mass index, the incidence of Schneiderian symptoms, or cumulative antipsychotic dose among the patient groups exhibiting ventricular enlargement, shrinkage, or no change in VV.

**Conclusion:**

Both enlargement and ventricular shrinkage are equally present in the early stages of schizophrenia. The newly discovered early reduction of VV in a subgroup of patients emphasizes the need for further research to understand its mechanisms.

## Significant outcomes

•Ventricular shrinkage is as frequent as ventricular enlargement in the early course of the disease.

•Ventricular enlargement is associated with the severity of negative symptoms.

**Limitations**

•MRI scans were taken at only two time points, which may not capture the full range of changes in ventricular volume that occur throughout the illness.

•Not all possible confounding factors that might affect VV changes (such as exposure to infectious agents, smoking, medical comorbidities, and polytherapy) were involved.

## Background

Schizophrenia is linked to a wide range of morphometric brain changes in white and gray matter [1, 2], with ventricular enlargement being one of the most recognizable macrostructural signatures [3, 4]. A recent meta-analysis of cross-sectional data has shown that this pattern is consistently observed in a significant proportion of chronic schizophrenia cases [5]. Studies have further demonstrated a correlation between the progressive reduction of gray matter and the progressive enlargement of the ventricles in the brain [6, 7].

However, the exact timeframe for these changes during the disease remains unclear. The phase preceding onset and the first four years after the illness onset are critical in schizophrenia, as they represent the period of the steepest gradient of progressive neuroanatomical brain changes [8, 9].

This anatomical deviation is noticeable in the early stages of the illness. The observation of ventricular enlargement in patients experiencing their first episode of schizophrenia (FES) was first reported two decades ago [10]. Although following studies have shown significant ventricular enlargement over time in first-episode schizophrenia patients [4, 11–13], there have also been conflicting results indicating no such enlargement in either first-episode or high-risk subjects [14], underscoring the need for additional research to reconcile these ambiguous findings.

A further important area of investigation is the link between ventricular volume and patient clinical outcomes. Studies in this area are scarce, with most being cross-sectional in design, underpowered, and primarily conducted on chronic patients. Crow [15] was the first to propose an association between ventricle enlargement in chronic schizophrenia and more severe negative symptomatology in his seminal work defining types I and II of the disease. This association has been subsequently confirmed in both cross-sectional and longitudinal studies [16–18]. Moreover, while earlier research suggested that ventricular enlargement is more prominent in first-episode psychosis patients with unfavorable clinical consequences [10, 19], recent studies have cast doubt on this association [20]. More robust and consistent findings from longitudinal studies have established a link between ventricle enlargement in first-episode patients and the severity of negative symptomatology [21–22].

Despite extensive research, understanding the etiology of ventricular volume changes in schizophrenia remains challenging due to the inherent heterogeneity of the disease. Schizophrenia is acknowledged as a complex syndrome characterized by diverse symptoms and trajectories [23]. Decades of investigation have indicated that schizophrenia is not a singular entity but a syndrome encompassing multiple distinct conditions [24–26]. It is imperative to reconsider the prevailing practice of employing a one-size-fits-all approach when comparing individuals with schizophrenia to controls. Given the disorder’s heterogeneity, it is essential to mitigate the confounding effects of diverse neurotypes before delineating boundaries between specific disease subtypes.

This study aims to identify diverse trajectories in ventricular volume dynamics during the early stages of the disease. If proven, it would underscore the notion of heterogeneity within the diagnostic category of schizophrenia, emphasizing the need for a more stratified approach in schizophrenia research. To achieve this goal, prospective morphometric data were collected via MRI at baseline (11.7 months after disease onset) and follow-up (one year later) in subjects with first-episode schizophrenia (FES). We hypothesized that patients may exhibit distinct ventricular volume change trajectories that correlate with clinical symptoms and functional outcomes, including PANSS-N severity, GAF scale severity, duration of untreated psychosis (DUP), the occurrence of first-rank Schneiderian symptoms, and body mass index (BMI) one year after the baseline visit.

## Methods

Patients and healthy controls originate from the prospective Early-Stage Schizophrenia Outcome study [27]. The healthy control subjects (HC) were sourced through advertisements, ensuring they shared similar sociodemographic characteristics with the First Episode Schizophrenia (FES) participants. Matching was meticulously done for each participant based on age and gender. To qualify as a control subject, stringent exclusion criteria were applied. These included: no personal history of psychiatric disorders or substance abuse, verified by the Mini International Neuropsychiatric Interview (M.I.N.I.) [28], and absence of psychiatric illness in immediate or extended family members. Additionally, both the control and FES groups were subject to further exclusion criteria, encompassing a range of medical and psychological conditions. These included any existing neurological disorders, a history of seizures, head injuries resulting in altered consciousness, intracranial hemorrhage, neurological sequelae, intellectual disabilities, a history of substance dependence, and any factors rendering MRI scanning unsafe or inappropriate.

The diagnosis of schizophrenia was established using a structured interview format, specifically the Mini International Neuropsychiatric Interview (M.I.N.I.) version 5.0.0 [28], conducted by a skilled psychiatrist. The study included individuals who had < 24 months of duration of psychosis and were 18–60 years old. We aimed to recruit participants at the early stages of illness to minimize the effects of disease and medications on brain structure. Exclusion criteria included a history of neurological or cerebrovascular disorders, substance use disorders, epilepsy, mental retardation, and any Magnetic Resonance Imaging (MRI) contraindications. Both inpatients and outpatients were recruited.

Demographic, clinical (Table 1), and neuroimaging data were collected at baseline (Visit 1, V1) and one-year follow-up (Visit 2, V2). Baseline psychopathology was evaluated with the PANSS [29] and GAF [30]. A composite score was obtained for negative symptoms using the sum of the seven items in PANSS (N1–N7 scores). We gathered Duration of Untreated Psychosis data at the first visit (V1). DUP was calculated as the time between psychosis onset and the start of antipsychotic treatment, with onset defined as one week or more of symptoms. We determined psychosis onset from medical reports, clinician referrals, or clinical assessments and updated the onset date as new information emerged during the study. Finally, we calculated the DUP value based on all available prospective data.

### MRI acquisitions

The study involved conducting MR examinations on two groups of participants, FES and HC, using either a 3T MR Siemens TRIO scanner (MRI Site 1, 101 subjects) or a 3T Siemens Prisma MAGNETOM MRI scanner from Siemens Medical Systems in Erlangen, Germany (MRI Site 2, 140 subjects). MRI scanning was performed on the same scanner for each subject at all visits to ensure methodological robustness, using consistent neuroimaging infrastructure to assess differences between visits. Additionally, inter-scanner reliability was evaluated by examining ten healthy controls at both MRI sites within two months. The analysis showed excellent correspondence between resulting anatomical volumes, with Pearson’s correlation coefficients between MRI Site 1 and 2 of *r* = 0.996 (*p* < 0.001) for both left and right lateral ventricles. Bland-Altman analysis revealed no substantial deviation or bias in the data obtained from either site. This high level of inter-scanner reliability enabled the combination of data from both sites, allowing for pooled analysis without the influence of any scanner-specific differences.

The study involved analyzing high-resolution T1-weighted 3D magnetization-prepared rapid gradient-echo (MP-RAGE) sequence images at two time points: baseline (V1) and mean 15.2 months afterward (V2) for both FES and HC groups. The FES and HC groups were matched in age and gender to the baseline patient’s characteristics. The MRI acquisition parameters were as follows: Site 1: TR/TI/TE = 2300/900/4.63 ms, voxel size 1 × 1 × 1 mm3; Site 2: TR/TI/TE = 2400/100/2.34 ms, voxel size 0.7 × 0.7 × 0.7 mm3.

MRI images were processed with the FreeSurfer software package [31]. FreeSurfer version 6.1 was used to process data from MRI Site 1, while version 7.1.1 was used for Site 2. For Site 1, which acquired data using older hardware resulting in lower quality and resolution (1 mm^3^), an operator extensively checked for errors using the FreeSurfer troubleshooting guideline, focusing on correcting skull stripping errors, control points in temporal regions, and white surface placement errors. Data of unsatisfactory image quality were excluded. Site 2, which used more up-to-date hardware resulting in higher resolution (0.7 mm3), was only inspected, and subjects with unsatisfactory image quality (primarily motion artifacts) were excluded. No manual interventions were performed.

Data of V1 and V2 were processed with the longitudinal stream of FreeSurfer [32]. Specifically, an unbiased within-subject template space and image is created using robust, inverse consistent registration. Processing steps, including tasks like skull stripping, Talairach transforms, atlas registration, generation of spherical surface maps, and parcellations, were subsequently initiated using shared data from the within-subject template. This approach markedly enhances both the reliability and statistical power of the analysis.

To accurately measure the volume of the lateral ventricles, we used the summation of specific anatomical regions based on previous research and established anatomical conventions [33]. These regions include the Left Lateral Ventricle, Left Inferior Lateral Ventricle, Left Choroid Plexus, Right Lateral Ventricle, Right Inferior Lateral Ventricle, and Right Choroid Plexus.

### Statistics

The study examined the proportion of first-episode schizophrenia in three groups based on ventricular volume changes (ventricular shrinkage /VS/, ventricular enlargement /VE/, and no change/NULL/). The χ2 test was used to compare the FES proportions among the groups. The study also analyzed the differences among the groups in demographic and clinical variables using the non-parametric Kruskal-Wallis test, with p-values adjusted for multiple comparisons using the Holm method [34]. The variables examined included age at baseline, gender, age of illness onset, cumulative dose of antipsychotics, duration of illness, duration of untreated psychosis, and body mass index.

The study utilized nonlinear mixed-effects models to analyze the dynamics of ventricular volume changes over time in each group. We utilized linear mixed models with nonlinear terms, employing the nlme function from the R package nlme. This approach, based on Lindstrom and Bates [35], allows for nested random effects, and accommodates within-group error correlations and unequal variances. Our model included the duration of untreated psychosis as a fixed effect to study its impact, with patient subjects as random effects to account for individual differences. We used a logistic curve to model the nonlinear relationship between the outcome and disease duration. Time and repeated measures were handled using a specific correlation matrix within the nlme framework, capturing the temporal structure and repeated observations in our data. This methodology was chosen for its robustness in representing the complexity of our data, ensuring reliable results.

Three different models were considered for each group: a constant model, a linear model, and an S-shaped model based on a logistic curve. The models were compared using log-likelihood, and the statistical package R, version 4.2.1 [36] was used for analysis. P-values less than 5% were considered statistically significant.

## Results

### Different trajectories in one-year ventricular volume changes

Initially, we evaluated the dynamics of changes in ventricular volume between baseline visit (V1) and after one year (visit 2, V2) in 113 patients (FES) and 128 healthy controls (HC, for demographic/clinical data, see Table 1).

**Table 1:** Overview of sociodemographic data of the study sample (*N* = 241)

	HC V1	HC V2	FES V1	FES V2
Sex, n = male/female	54 / 74		67 / 46	
Age in years	M = 29.20; sd = 7.54	M = 30.49; sd = 7.60	M = 28.09; sd = 7.03	M = 29.30; sd = 7.00
Years of education	M = 17.07; sd = 3.13	M = 17.72; sd = 3.29	M = 14.37; sd = 3.14	M = 14.83; sd = 3.24
BMI	M = 23.40; sd = 3.58	M = 23.48; sd = 3.45	M = 24.75; sd = 4.77	M = 27.00; sd = 5.50

**Legend**: HC = healthy controls; V1 = baseline visit; V2 = visit one year after the baseline; FES = first episode of schizophrenia patient group; BMI = Body Mass Index;; M = median; sd = standard deviation

In the sample of patients, the median age of psychosis onset was 28 years (median = 27.9, sd = 7.7); the duration of illness at V1 was 11.7 months, with a median duration of untreated psychosis of 6 months. Instead of directly comparing FES to healthy controls, the study first analyzed normative data on ventricular volume changes in a sample of HC matched to FES for age and sex. The normative sample showed a median one-year VV change of 0.009 ccm, with upper and lower bounds calculated as 0.025% and 0.975% quantiles (-0.066; 0.125 ccm). These numerical cut-offs allowed for parsing pooled FES + HC samples into three different groups based on the individual values of V1-V2 change in VV falling within the specified bounds: no VV change (NULL group), ventricular shrinkage (VS group), or ventricular enlargement (VE group).

The VE group consisted of 21 FES (18.6% of all FES) and 4 HC (3% of all HC), respectively. Most of the sample showed no significant change of VV during the one-year follow-up (NULL group; 73 FES, 64.6% of all FES; 120 HC, 94% of all HC), while the rest of the sample exhibited marked ventricular shrinkage (VS group; 19 FES, 16.8% of all FES; *n* = 4 HC, 3% of all HC). A χ2-test revealed statistically significant differences in the proportion of FES assigned to these groups (*p* = 0.0000). Significantly more FES were assigned to VE (*p* = 0.0001) and VS groups (*p* = 0.0001), respectively, compared to HC. There was no significant difference in the proportional contribution of FES/HC within the NULL group. These results suggest that FES have a higher risk of ventricular enlargement or shrinkage than HC, as measured by VV changes over one year.

**Fig. 1:**
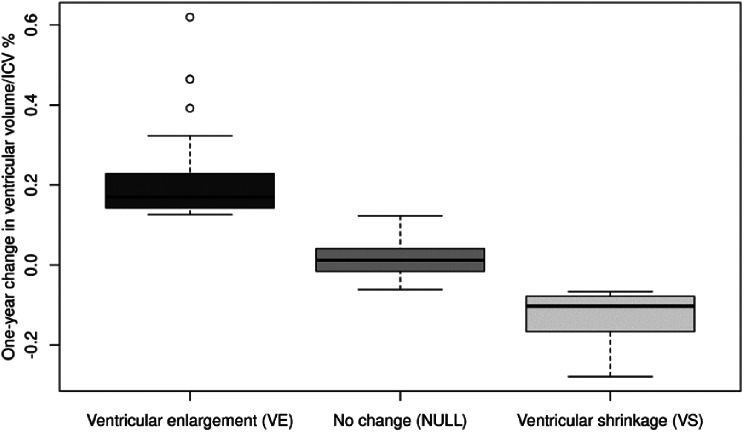
One-year change in ventricular volume in individual pooled groups (FES + HC)

The median annualized ventricular volume change in patients with FES differed by group: VE group: 14.1% (95%CI: 5.4 to 33.1), VS group: -9.4% (95%CI: -23.7 to -2.4), and NULL group: 2.0% (95%CI: -5.6 to 11.4). In contrast, healthy controls had the following median annualized ventricular volume change: VE group: 5.5% (95%CI: 3.8 to 7.1), VS group: -5.6% (95%CI: -7.5 to -4.6), and NULL group: 0.5% (95%CI: -5.9 to 6.5). Figure 1 provides additional details.

Among patients with FES, there were no significant differences in sex, duration of illness, age of illness onset, duration of untreated psychosis, the proportion of patients with first-rank Schneiderian symptoms, BMI [37], or cumulative dose of antipsychotics in chlorpromazine equivalents between the VE, NULL, and VS groups (Kruskal-Wallis test with corrections for multiple comparisons by Holm method). However, at one-year follow-up (Visit 2, V2), the VE group had significantly increased PANSS-negative symptoms compared to both the NULL (*p* = 0.011) and VS (*p* = 0.018) groups. Tables 2 and 3 provide descriptive data for the three ventricular change groups, separated into patient and control groups.

**Table 2:** Average values and standard deviations (in brackets) for relevant variables for groups split by ventricular change type

	Patients			Controls		
	VE	normal	VS	VE	normal	VS
*N*	21	73	19	4	120	4
Gender (m / f)	12 / 9	45 / 28	10 / 9	1 / 3	51 / 69	2 / 2
Age at V1	28.47 (7.24)	28.86 (6.95)	24.97 (6.71)	28.25 (6.19)	29.12 (7.38)	32.40 (16.72)
Age at V2	29.80 (7.04)	30.05 (6.94)	26.12 (6.82)	30.07 (5.27)	30.40 (7.46)	33.58 (16.58)
Visit time interval (months)	16.02 (6.26)	14.25 (4.50)	13.83 (1.90)	21.80 (13.00)	15.28 (4.74)	14.19 (2.63)
Years of education at V1	14.62 (4.43)	14.40 (2.85)	13.92 (2.83)	17.25 (2.08)	17.03 (3.22)	18.00 (1.41)
Years of education at V2	15.50 (4.63)	14.47 (2.88)	14.56 (3.24)	18.75 (1.73)	17.65 (3.38)	18.00 (1.41)
BMI at V1	23.76 (3.71)	25.17 (4.96)	24.04 (5.34)	22.82 (2.92)	23.37 (3.57)	24.67 (6.07)
BMI at V2	25.58 (5.03)	27.32 (5.55)	27.16 (6.16)	21.90 (2.36)	23.52 (3.42)	23.94 (5.11)

**Table 3:** Illness severity data for patients split by ventricular volume change (average values and standard deviations; *N* = 113)

	Normal	VS	VE	p-value	Adjusted p-value
Age of first symptoms	27.77 (6.83)	23.90 (6.50)	27.72 (7.45)	0.0726	0.7109
Duration of untreated psychosis (months)*	7.03 (14.08)	3.05 (7.53)	6.74 (7.58)	0.1220	0.7320
Chlorpromazine equivalent V1*	379.37 (244.50)	376.21 (196.06)	344.40 (131.19)	0.8331	1.0000
Chlorpromazine equivalent V2*	264.82 (191.41)	311.57 (161.71)	315.80 (212.86)	0.3744	1.0000
PANSS - P V1*	12.19 (3.99)	12.74 (3.51)	14.65 (4.72)	0.0821	0.7109
PANSS - N V1	17.06 (4.93)	19.79 (7.76)	17.55 (5.38)	0.5453	1.0000
PANSS - G V1	30.26 (7.58)	35.79 (8.43)	34.00 (7.30)	0.0161	0.2099
PANSS total V1*	59.51 (13.82)	68.32 (17.31)	66.20 (14.66)	0.0711	0.7109
PANSS - P V2*	9.89 (3.57)	9.37 (3.18)	11.76 (4.93)	0.1297	0.7320
PANSS - N V2*	14.55 (5.42)	13.89 (5.65)	17.95 (5.31)	0.0327	0.3903
PANSS - G V2*	25.73 (6.94)	25.26 (6.44)	31.90 (8.79)	0.0127	0.1773
PANSS total V2*	59.51 (13.82)	68.32 (17.31)	66.20 (14.66)	0.0711	0.7109
GAF V1	65.27 (15.24)	58.22 (16.96)	60.38 (18.59)	0.2282	0.9126
GAF V2*	75.36 (14.93)	75.84 (12.53)	66.86 (14.17)	0.0325	0.3903

*Note*: *=Shapiro-Wilk test indicated violation of assumption of normality; PANSS - Positive and Negative Syndrome Scale (N - Negative symptoms; P - Positive symptoms; G - General psychopathology); GAF - Global Assessment of Functioning scale

### Ventricular volume dynamics in three groups with different trajectories

**Fig. 2:**
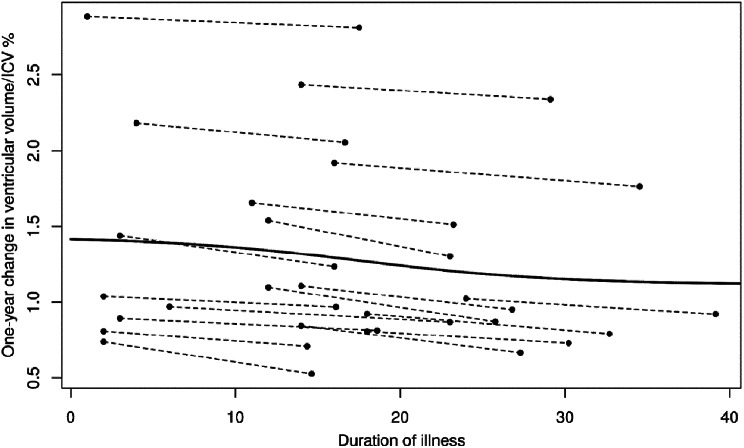
Changes in total ventricles/ICV % depending on the duration of illness, shrinkage group, S-curve model

**Fig. 3:**
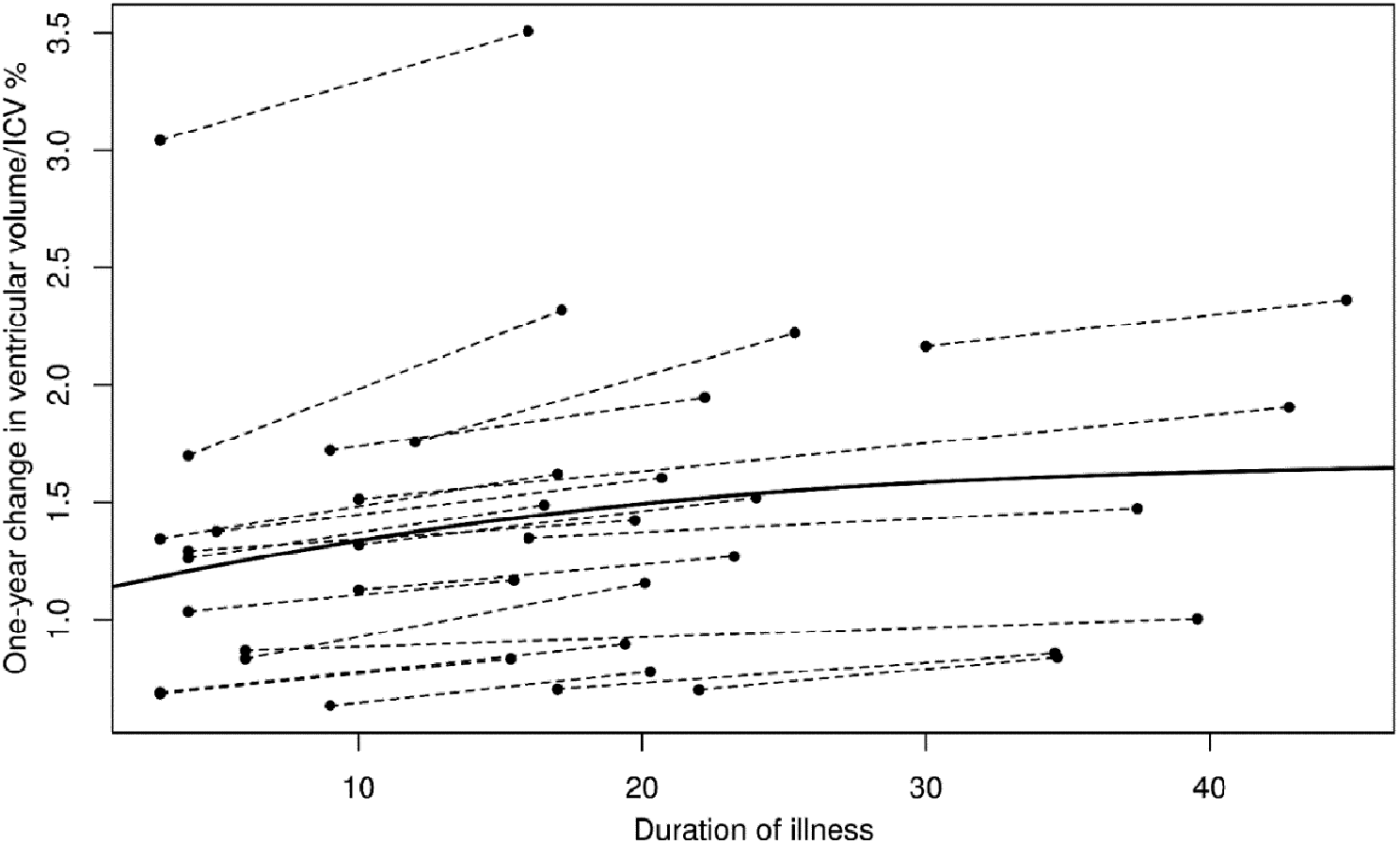
Changes in total ventricles/ICV % depending on the duration of illness, enlargement group, S-curve model

We utilized a longitudinal dataset from patients with FES to examine ventricular volume dynamics in three distinct VV change groups. Individual VV measurements at baseline (V1) and follow-up (V2) were plotted on the x-axis, with the duration of illness shown on the y-axis in Figs. 2 and 3. We analyzed the VV dynamic characteristics within separate patient groups: VE, VS, and NULL. We compared three different models - a constant model (CM), a linear model (LM), and an S-shaped model (SM) - to identify the best goodness-of-fit for each group. We found that the S-curve model was the best fit for the VE and VS groups, with VV plateauing in both groups approximately 20 months after illness onset (CM < LM, *p* = 0.00001; LM < SM, *p* = 0.003 for the VE group; CM < LM, *p* < 0.0001; LM < SM, *p* < 0.0002 for VS group). The NULL group also showed the best fit with the S-curve model (CM < LM, *p* < 0.0001; LM < SM, *p* = 0.005), although an initial bland increase in VV leveled off a few months after illness onset suggested the possibility of noise in the data.

## Discussion

Only a few longitudinal studies have analyzed lateral ventricular volume dynamics in a sample of early-stage schizophrenia subjects [38]. Our analysis contributes to the current understanding of early changes in lateral VV in a relatively large sample of first-episode schizophrenia patients.

We derived our method of categorizing patients based on changes in ventricular volume—into ventricular shrinkage (VS), ventricular enlargement (VE), and no change (NULL)—from cut-offs within a 95% confidence interval for one-year ventricular volume (VV) changes observed in a normative database of healthy controls.

While previous studies have reported canonical ventricular enlargement in schizophrenia, our findings reveal a novel and surprising observation. We observed a similar proportion of patients with significant ventricular reduction (VS group, 16.8%) as those with significant ventricular enlargement (VE group, 18.6%) compared to controls.

Furthermore, the study highlights the dynamic nature of VV changes in schizophrenia. We found that the VE group exhibited a significant 14% annual increase in VV, comparable to the ventricular enlargement rate reported in Alzheimer’s disease [39]. This observation indicates that variations in VV can uncover notable structural dynamics in the early stages of schizophrenia.

The key finding of this study challenges the prevailing belief that ventricular enlargement is the primary morphological pattern in the early stages of the disease. Instead, we have demonstrated that ventricular shrinkage is similarly frequent and, thus, a significant feature in this phase. Only two MRI longitudinal studies with small sample sizes reported decreased VV in FES patients. Specifically, Puri et al. found that almost half of their patient sample (6 out of 14) showed a percentage VV decrease or increase that exceeded two standard deviations compared to healthy controls during the average eight months of follow-up [40]. Similarly, DeLisi et al. found that out of 24 first-episode patients who completed a 2-year follow-up period, three patients displayed a 20% decrease in VV [41]. These results indicate that the changes in ventricular volume during the early stages of schizophrenia are not unidirectional and that the path of change may vary between individuals.

Our study also revealed that changes in ventricular volume correlate with clinical symptoms. Consistent with prior research [6, 16, 42–43], the ventricular enlargement was associated with increased negative symptoms in our sample. However, the data did not reveal a clear link between ventricular shrinkage in the VS group and any specific clinical pattern, suggesting that the mechanisms behind VV dynamics in schizophrenia may be complex and multifactorial.

In summary, the conflicting changes in longitudinal VV in early-stage schizophrenia, along with a considerable number of cases showing no alteration in VV size over time, could clarify why some prior studies did not find any significant changes in the size of the lateral ventricles during this stage of the illness [44].

The neurobiological mechanisms underlying these dynamic VV alterations remain unclear. Over 300 MRI studies have confirmed the consistent finding of lateral ventricular enlargement in schizophrenia, which is typically around 25% larger by volume in chronic cases [45]. Previous research on schizophrenia has predominantly focused on the factors contributing to ventricular enlargement, yet the findings have remained inconclusive over the decades [38, 46].

Meanwhile, due to a lack of evidence, the pathophysiological mechanisms of ventricular shrinking in this population have yet to be discussed. According to the Monro-Kellie doctrine, the total volume inside the skull remains constant. This means that if the volume of one component, such as brain tissue or blood, increases, the volume of the other component, cerebrospinal fluid (CSF), must decrease. Ventricular shrinkage may occur due to diverse pathological changes in brain tissue volume, impairing the movement of intracerebral fluids within an intracranial system comprising interconnected compartments. In other words, ventricular volume decrease may primarily be based on fluid mechanical principles. Various factors, including inflammatory processes, craniocerebral injuries, endocrine and metabolic changes, or neurodegeneration, can cause the accumulation of fluids within the brain [47].

The Monro-Kellie doctrine suggests that microedema can cause a shift in brain compartment volumes, resulting in a detectable decrease in ventricular volume [48–49]. This could explain the observed ventricular shrinkage in early-stage schizophrenia, as there is evidence of inflammation during this phase [50]. Inflammation has been shown to lead to the accumulation of water within the brain due to various pathophysiological processes [51–52]. Additionally, recent studies have confirmed the presence of specific inflammatory subtypes in antipsychotic-naive patients with first-episode schizophrenia, which are associated with cortical expansion [53]. In parallel, chronic schizophrenia patients with elevated inflammatory markers have displayed evidence of cortical swelling [54]. However, neither of these studies reported ventricular shrinkage. Interestingly, a diffuse MRI acquisition has confirmed increased extracellular free water content in the brain during the first years of psychosis, but this appears to vanish throughout the illness [55–56]. In summary, the observed ventricular shrinkage in certain cases of schizophrenia may be explained by dynamic alterations in extracellular free water content in the brain during the early stages of the illness.

An important aspect of this finding is the absence of reported ventricular shrinkage in cases of chronic schizophrenia. This suggests that brain tissue swelling, whether in white or gray matter or both, could be an initial response to an acute stressor, likely of inflammatory origin, during the prodromal and early stages of the disease. This response may lead biphasically to cortical gray matter loss, followed by ventricular normalization or enlargement. A similar two-step trajectory of microstructural and macrostructural changes has been observed in the early stages of Alzheimer’s disease, suggesting the non-specificity of this degenerative pathway [57].

The present study has limitations that should be considered when interpreting the results. The neurobiological homogeneity of schizophrenia remains a pending concern within the scientific and clinical community. To address this, we selected first-rank Schneiderian symptoms as a marker for the core schizophrenia phenotype in post hoc analyses [58]. There was no significant difference in the proportion of subjects with first-rank Schneiderian symptoms across the three groups exhibiting different ventricular dynamics. Overall, the prevalence of first-rank symptoms was high in all three groups, with VE at 85%, NULL at 83%, and VS at 79%. Secondly, the limitations include the lack of a validation sample and the exclusion of factors such as exposure to infectious agents, smoking, medical comorbidities, polytherapy, and sleep issues. However, major confounding factors such as age, duration of illness, duration of untreated illness, antipsychotic dose, and BMI were controlled for. Additionally, the study relied on MRI scans taken at only two time points, which may not capture the full range of changes in ventricular volume that occur throughout the illness. Future studies should aim to address the aforementioned limitations and explore these areas further to better understand the observed ventricular changes in early-stage schizophrenia.

## Conclusion

This study is one of the largest longitudinal morphometric MRI investigations of ventricular volume dynamics in early-stage first-episode schizophrenia. The findings challenge the conventional notion of a unidirectional VV expanding trajectory in schizophrenia, as ventricular shrinkage and enlargement were found to occur with similar frequency during this phase, underscoring the heterogeneity of the disease. The observed reduced ventricular volume during the early stages of illness raises questions about the underlying neurobiology, highlighting the need for further research to elucidate the mechanisms involved.

## Data Availability

The data supporting this study’s findings are available from the corresponding author upon request.

## Abbreviations

FES: first-episode schizophrenia
HC: healthy controls
VV: ventricular volume
DUP: duration of untreated psychosis
BMI: body mass index
MRI: Magnetic Resonance Imaging
MP-RAGE: magnetization-prepared rapid gradient-echo
VS: ventricular shrinkage
VE: ventricular enlargement
NULL: no change
PANSS: Positive and Negative Syndrome Scale
GAF: Global Assessment of Functioning scale
CM: a constant model
LM: a linear model
SM: and an S-shaped model
CSF: cerebrospinal fluid
